# Clark County Medical Society

**Published:** 1868-01

**Authors:** F. R. Payne, J. D. Mitchell

**Affiliations:** Pres.; Sec’y.


### CLARK COUNTY MEDICAL SOCIETY.

This Society met on the 6th day of November, 1867, at the office of Dr. Gard, Martinsville. The numbers present fully appreciated the hospitality of Drs. Gard and McNary.

Dr. J. D. Mitchell, of Darwin, read a paper on "Shoulder Presentation." Drs. S. Juniper and D. O. McCord, of York, were consulting physicians.

At the time they visited the patient, the waters were evacuated, and the tonic contraction complete, consequently cephalic version was impossible, and it was also impossible to bring down the foot. In this condition, the only remedy seemed to be decapitation, which was, by mutual consent, performed successfully by Dr. Mitchell. The body of the child was then delivered without any difficulty, and then the head. The patient had a rapid recovery.

This report led to a spirited discussion on shoulder presentation, in which all the members participated.

Dr. F. R. Payne read a lengthy paper on the "Philosophy of Practical Medicine." He fully exposed the falacy of all theories that could not be supported by experience and careful tests.

The thorough study of diseases, and the effects of their modifiers, cannot fail to lead to important practical results.

Dr. Spencer read a paper on "Carcinomatous Diseases." For all practical purposes, he contended that it was only necessary to be familiar with three varieties—scirrhus, encephaloid, and colloid. These varieties he regarded as distinct, and if not removed, must, in all cases, result in death. The great question, he said, was, can cancer be cured? For the last twenty years he had spent much time in the study of this disease, and had thoroughly tested the value of many remedies. In some cases the knife may effect a radical cure; but if the disease is deep-seated, this remedy only brings temporary relief.

For many years he has abandoned the use of the knife, and relies upon sulphate of zinc locally applied to the tumor, in a fine powder. If the system is effected with the virus, he uses tonics and other remedies, so as to thoroughly prepare the system before the application of the medicine.

Dr. F. R. Payne offered the following resolutions, which were unanimously adopted :—

*Resolved*, That the members of this Society heartily approve of the recent action of the convention of delegates from the medical colleges of the United States, held in Cincinnati last May, which was emphatically endorsed by a unanimous vote of the American Medical Association, urging an improvement in the whole work of medical education in this country.

*Resolved*, That we are fully satisfied that it would add greatly to the honor and usefulness of our profession if all of our medical colleges would adopt a possitive standard of preliminary education—the long terms of collegiate instruction—a systematic and successive order of study, with chemical instruction in public hospitals.

Drs. Gard and McNary gave a very interesting history of epidemic diphtheria as it prevailed in their locality during this fall. The treatment found most successful was very simple, and consisted in olive oil, chlorate, potass, and honey, mixed, and given freely. They also, after the first few days, gave mul. tinct. ferri. Under this treatment, nearly all the cases got well.

The members present reported that the last summer and fall had been remarkably healthy. We have had but very few cases of autumnal fevers. This was, doubtless, owing to the extreme drought in this part of the country.

The Society then adjourned, to meet in Marshall, Illinois, on the 1st Wednesday of January, 1868.

F. R. PAYNE, *Pres.*

J. D. MITCHELL, *Sec'y.*

